# Dentists in the Army and Navy

**Published:** 1899-08

**Authors:** Herbert H. Shapard

**Affiliations:** Austin, Texas


### Dental Department.

### Dentists in the Army and Navy.

BY HERBERT H. SHAPARD, D. D. S., AUSTIN, TEXAS.

Having been requested by the editor of the JOURNAL and several of my fellow practitioners to give my views and experiences in regard to the practice of dentistry in the army, I will endeavor to write a synopsis of what I learned and observed during a practice of seven months while in the service.

At the outbreak of our late war with Spain I was a member of the local military company (The Governor's Guard), and felt it my duty to offer my services to my country, which I did, with the company, on May 3, 1898. We were assigned to the First Texas regiment, and spent eleven months in the service, during which time we were not exposed to the Spanish shot and shell, but I claim the honorable distinction of going up against "Army grub" for eleven long months, and being one, of only two in the company, whose name did not appear on the sick report.

We were in camp at Mobile, Ala., during the month of June; from there we went to Miami, Florida, where we spent about six weeks; every day during this period one man or more would come to me and ask me to send after my instruments, as they had dental work to be done. Our Colonel, Mabry, offered to appoint me regimental dentist (which of course, was an honorary appointment, no provision having been made for such). After due deliberation, I concluded to send after them, and received them at Jacksonville, Florida, about the latter part of August, when I proceeded to open my military dental office in a box house about twelve feet square, which was built for a company commissary. After putting a window sash in the roof, I had a very good office, considering the circumstances. I then proceeded with the aid of a hatchet, saw and pocket knife, to construct a chair out of material consisting of a few pieces of rough one by one and a half and a hard tack box, upholstering it with a piece of red flannel and some moss. This done, I made a table on which to place my instruments. After getting everything in position I was ready for business, although my surroundings were not such as you would expect to find in a regular office. For instance, instead of occupying comfortable rockers while waiting for my services, my patients were expected to sit on a sack of potatoes, or anything that was more convenient, and, instead of such sanitary surroundings as fountain spittoons, etc., patients were expected to expectorate on the floor of mother earth.

My services were in great demand, and besides my regular practice, I was frequently called upon by the surgeons to visit the hospital and treat dental disorders of patients confined therein. I was often awakened from my peaceful slumbers at all hours of the night and requested to get up and treat cases of odontalgia, alveola abscesses, etc.; in fact, my services were demanded from the colonel down to the company cook, and, during the three months we spent in Cuba, by members of the Second Louisiana, Second South Carolina, and Third Nebraska regiments, and on several occasions by the hospital surgeons themselves. I can endorse everything my old classmate, Dr. Schamberg, says in his article in the June number of the *International Dental Journal,* in which he very properly and wisely advocates the appointment of one hundred dentists in the regular army in the field.

I can not see why it is not considered just about as important to have dentists in the army and navy as to have physicians. No one will dispute the fact that in order to maintain good health the organs of mastication must be kept in good condition. In order to properly masticate *army rations* every organ connected with the oral cavity should be fully developed and in a thoroughly healthy condition. That the contrary was the fact, I was a witness to daily. I have had soldiers present themselves to me for treatment whose mouths were in a horrible condition—teeth decayed and filled with decomposed organic matter and food; gums often badly affected with pyorrhea alveolaris, chronic abscesses discharging foul matter into the mouth, this in turn swallowed into the stomach, the thought of which, in itself, is enough to make a man with an iron constitution or a healthy person sick. A great many would tell me that their teeth were in good condition when they enlisted in the army, and it is reasonable to suppose that they were telling the truth, for it is one of the regulations and requirements in order to stand the physical examination to enter the army that their teeth shall be in good order. I knew of several men who were refused admission on account of their teeth not being in proper condition. Now, this being the case, why is it not essential to *keep* their teeth in good condition?

I have heard it argued that to establish a branch of dentistry in the service would be too great an expense to the government. I think it could be done at a very small expense. Every regiment, I believe, is supposed to have a major-surgeon, two assistant surgeons, and generally a good hospital steward, who attends to most of the minor ailments of the men. Why should not a dentist be appointed in the place of one of the assistant surgeons? I am of the opinion that it would prevent enough sickness to fully justify the change, and would not increase the expense of the government, and I will venture the assertion that the dentist would be the busiest man of the three.

When in a foreign country it is sometimes rather difficult to secure the services of a competent dentist. In search of a dental depot while in Havana, I visited several dental offices. I did not see a dentist in my rounds whom I would consent to have operate in my mouth. One of them looked as if his own teeth were about to drop out of his mouth from the ravages of pyorrhea alveolaris.

To show how much a dentist is appreciated in a foreign country by our soldiers, I will state two little incidents which happened in my practice while in Havana. One day the major-surgeon of our regiment came to me and said that he would like for me to go over to the second division hospital and extract a tooth for one Dr ——, who was on duty there and suffering intense pain. On examination I found it to be a lower right third molar screwed up against the ramus of the jaw, and about as difficult to extract as any I ever undertook. After extracting it, I found that the roots were curved forward and were locked between the roots of the twelve-year molar. When asked how much my charges were, and I replied one dollar, he remarked that he had been suffering intense pain for about ten days, and during that period had lived on mush and milk, and handing me two dollars, said that it was d—d cheap at that. About thirty minutes after reaching my tent, again the same major-surgeon came up with an officer of the Second Louisiana regiment and informed me that the gentleman was suffering with one of his teeth and would like to have it extracted. On examination I found it to be an upper right twelve-year molar in a bad state of decay, and had an abscess on the posterior bucal root. After extracting it I was handed a five dollar bill and told to take half of it, but not having the exact change, I handed him three dollars, whereupon he handed me back one dollar with the remark, that it was worth three dollars to him. I could go on and cite numerous such instances, but these two are sufficient.

For the benefit of Dr. Crouse I will state that in the army I was never annoyed by competition, and would advise him to enlist. I

could write a great deal on this subject, but as I am limited to space will close by saying, that I hope Congress will be made to see the necessity of providing for dentists in the army in the near future.

